# Phytochemical Profiling and Pharmacoinformatics Reveal Proliverenol from *Phaleria macrocarpa* as a Multi-Target Hepatoprotective Modulator of MAFLD

**DOI:** 10.3390/ph19030491

**Published:** 2026-03-17

**Authors:** Fahrul Nurkolis, Aida Dama, Era Gorica, Antonello Santini, Raymond Rubianto Tjandrawinata

**Affiliations:** 1 Master of Basic Medical Science, Faculty of Medicine, Universitas Airlangga, Surabaya 60131, Indonesia; 2 Medical Research Center of Indonesia, Surabaya 60281, Indonesia; 3 Institute for Research and Community Service, State Islamic University of Sunan Kalijaga (UIN Sunan Kalijaga), Yogyakarta 55281, Indonesia; 4 Department of Pharmacy, Faculty of Medical Sciences, Albanian University, 1017 Tirana, Albania; 5 Department of Cardiac Surgery, Faculty of Medicine, University Hospital Zürich, University of Zürich, Wagistrasse 12, 8952 Schlieren, Switzerland; 6 Department of Pharmacy, University of Napoli Federico II, Via Domenico Montesano 49, 80131 Napoli, Italy; 7 School of Bioscience, Innovation and Technology, Atma Jaya Catholic University of Indonesia, Jakarta 12930, Indonesia; 8 Dexa Laboratories of Biomolecular Sciences, Dexa Medica, Industri Selatan V PP-7, Jababeka 2, Cikarang 17550, Indonesia

**Keywords:** Proliverenol, *Phaleria macrocarpa*, MAFLD, network pharmacology, metabolomics, molecular docking

## Abstract

**Background:** Metabolic dysfunction-associated fatty liver disease (MAFLD) is a highly prevalent chronic liver disorder driven by complex metabolic, inflammatory, and oxidative mechanisms with no effective pharmacological therapy currently available. Although the multi-target natural product Proliverenol, derived from *Phaleria macrocarpa* pericarp, has shown hepatoprotective potential in preclinical and early clinical studies, its molecular mechanisms in MAFLD remain unclear. **Objective:** This study aimed to elucidate the multi-target hepatoprotective mechanisms of Proliverenol in MAFLD by integrating untargeted phytochemical profiling, network pharmacology, and molecular docking approaches. **Methods:** Untargeted LC–HRMS/MS analysis was performed to characterize the phytochemical composition of Proliverenol (Veprolin™). Identified compounds were subjected to target fishing, followed by protein–protein interaction (PPI) network construction, cluster analysis, and functional enrichment (GO and KEGG). Key MAFLD-related targets were further validated using molecular docking against major signaling proteins implicated in inflammation, apoptosis, and metabolic regulation. **Results:** Fourteen bioactive compounds were annotated, dominated by flavonoids and organic acids, including several phenolic acid derivatives, with phalerin as the most abundant constituent. Network pharmacology identified overlapping targets between Proliverenol, MAFLD, and hepatotoxicity, forming a highly interconnected PPI network. Functional enrichment revealed significant involvement in apoptosis regulation, inflammatory signaling, oxidative stress response, lipid metabolism, and insulin resistance pathways. Molecular docking demonstrated strong binding affinities of several Proliverenol constituents—particularly cucumerin B, artoindonesianin P, and vitexin 2″-p-hydroxybenzoate—toward key targets including PTGS2, SIRT1, GSK3B, RELA, and MCL1, with affinities comparable to or exceeding those of reference drugs. **Conclusions:** Proliverenol exerts hepatoprotective effects through coordinated multi-target modulation of inflammatory, metabolic, and apoptotic pathways relevant to MAFLD. While these findings provide mechanistic insights based on integrative metabolomics and computational analyses, the absence of direct experimental validation represents an important limitation. Therefore, further in vitro, in vivo, and clinical investigations are warranted to confirm the predicted molecular interactions and therapeutic relevance.

## 1. Introduction

Metabolic Dysfunction-Associated Fatty Liver Disease (MAFLD), formerly known as non-alcoholic fatty liver disease (NAFLD), has become a major global health concern [1,2,3]. Epidemiological studies indicate that MAFLD affects roughly one quarter of the adult population worldwide [4]. In Asia and other regions, prevalence rates on the order of 25–30% have been reported, reflecting the condition’s status as one of the most common chronic liver diseases in the modern era [5]. MAFLD is closely linked to the rising incidence of obesity and metabolic syndrome [6]. It encompasses a spectrum ranging from simple hepatic steatosis to non-alcoholic steatohepatitis (NASH), the latter characterized by inflammation and hepatocellular injury that can progress to fibrosis, cirrhosis, and even hepatocellular carcinoma [4]. Despite its growing prevalence and serious complications, effective pharmacological therapies for MAFLD are still lacking [7]. Currently, clinical management relies mainly on lifestyle interventions (dietary changes and weight loss) and control of metabolic comorbidities, as no drugs have been approved specifically for MAFLD [8]. This therapeutic gap, combined with the complex, multifactorial pathogenesis of MAFLD—involving insulin resistance, excess lipid accumulation, oxidative stress, and inflammatory cascades—underscores the need for novel treatment strategies that can target multiple pathogenic pathways simultaneously [9].

In the search for multi-target therapeutic options, increasing attention has turned toward natural products and botanical extracts with hepatoprotective potential [10]. Traditional herbal remedies often contain a spectrum of bioactive compounds that can exert antioxidant, anti-inflammatory, and metabolic regulatory effects in parallel [11,12,13]. *Phaleria macrocarpa* (Scheff.) Boerl. (*P. macrocarpa*), commonly known as Mahkota Dewa, is one such natural product of interest in liver disease management [14]. This medicinal plant, native to Southeast Asia, has a history of use in folk medicine for treating various ailments, including cancer, allergies, and diabetes [15]. Phytochemical investigations have revealed that *P. macrocarpa* fruits are rich in diverse bioactive constituents, such as glycosides (e.g., mahkoside A), flavonoids (e.g., mangiferin and kaempferol-3-O-β-glucoside), fatty acids, and other compounds [16]. Notably, extracts of *P. macrocarpa* have demonstrated multiple pharmacological activities relevant to liver health—including pronounced antioxidant and anti-inflammatory effects—and have shown hepatoprotective efficacy in preclinical studies [16]. These findings suggest that *P. macrocarpa*’s complex phytochemical profile may confer a capacity to modulate the various pathological processes underlying MAFLD.

Building on the therapeutic potential of this herb, a standardized bioactive fraction derived from *P. macrocarpa*, known as Proliverenol (commercially formulated as Veprolin™), has been developed as a candidate hepatoprotective agent. Proliverenol is a standardized phytochemical fraction isolated from the dried fruit pericarp of *P. macrocarpa*, characterized by a high concentration of its key bioactive constituents. Preclinical studies have provided a scientific rationale for exploring Proliverenol in MAFLD. In cellular models of liver injury, Proliverenol was shown to enhance hepatocyte survival by up-regulating DNA repair mechanisms and down-regulating pro-apoptotic and pro-inflammatory signaling pathways (including the NF-κB/TNF-α/caspase-8 cascade), thereby preventing leakage of liver enzymes like ALT [17]. Consistent with these in vitro findings, in vivo studies have demonstrated that Proliverenol administration can attenuate liver damage in toxin-induced injury models. For example, in a carbon tetrachloride-induced fibrosis model, *P. macrocarpa* extracts (Proliverenol) reduced hepatic oxidative stress and inflammatory cytokine levels, resulting in significantly less fibrogenesis and improved liver histology [18]. Similarly, other experimental studies reported that Proliverenol treatment mitigates liver inflammation and fibrosis via its antifibrotic and anti-inflammatory actions [19]. Taken together, this evidence highlights Proliverenol as a promising multi-target modulator of MAFLD, capable of intervening in the disease’s diverse pathological pathways (such as lipid accumulation, inflammation, and fibrogenesis) to protect the liver.

To fully elucidate the mechanistic basis of Proliverenol’s hepatoprotective effects, the present study adopts an integrative research approach combining untargeted phytochemical profiling, network pharmacology, and molecular docking analyses. Untargeted metabolomic profiling (phytochemical analysis) is employed to comprehensively characterize the chemical constituents of Proliverenol, thereby identifying the spectrum of phytochemicals that could contribute to its biological activity. Network pharmacology, grounded in systems biology, is then utilized to predict and analyze the interactions between these multiple phytochemicals and various biological targets/pathways related to MAFLD. This approach allows construction of a compound–target network to elucidate how Proliverenol’s components might collectively influence key signaling pathways in metabolic dysfunction and fatty liver disease [20]. Notably, network pharmacology has emerged as a powerful tool for understanding multi-component herbal medicines, revealing therapeutic mechanisms that involve numerous targets rather than a single molecular hit [20]. Finally, molecular docking studies are integrated to validate the binding of key bioactive compounds from Proliverenol with specific protein targets implicated in MAFLD pathogenesis. By simulating ligand–protein interactions at the molecular level, docking analysis provides structural insights into the affinity and mode of binding of Proliverenol’s constituents to their putative targets. Through this multi-pronged methodology, our study offers a holistic perspective on how Proliverenol exerts its hepatoprotective effects. We aim to delineate the network of pharmacological actions triggered by Proliverenol, thereby shedding light on its multi-target mechanism of action in MAFLD and supporting the potential development of Proliverenol as an effective, mechanism-based hepatoprotective therapy.

## 2. Results

### 2.1. Phytochemical Profiling of Proliverenol

Untargeted LC–HRMS/MS analysis successfully characterized the phytochemical composition of Proliverenol (Veprolin™). A total of fourteen compounds were annotated with high mass accuracy (Δ mass < ±1 ppm) and reproducible retention times across duplicate analytical runs, confirming analytical robustness. The identified compounds comprised diverse chemical classes, including flavonoids, organic acids (including phenolic acid derivatives), nucleosides, and terpenoids (Table 1).

Among these, phalerin was the most abundant constituent, exhibiting the highest peak area (4.585 × 10^9^), followed by vitexin 2″-p-hydroxybenzoate, azelaic acid, and succinic acid. Several bioactive flavonoids, such as vitexin, artoindonesianin P, and cucumerin B, were also detected, supporting the complex polyphenolic nature of Proliverenol. The presence of metabolic intermediates (gluconic acid, succinic acid, 4-oxoproline) and signaling-related metabolites (adenosine, kynurenic acid) suggests potential multitarget biological activity relevant to hepatic metabolism and cellular stress responses.

### 2.2. Overlapping Target Identification and Protein–Protein Interaction Analysis

**Figure 1:**
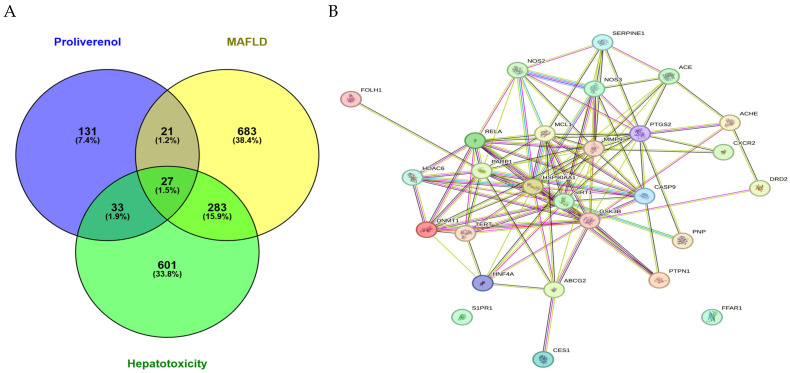
Venn diagram analysis of Proliverenol vs. MAFLD vs. Hepatotoxicity (**A**) and Protein–Protein Interaction (PPI) using the STRING database (**B**).

To elucidate the molecular relevance of Proliverenol in MAFLD and hepatotoxicity, a Venn diagram analysis was performed to identify overlapping targets among Proliverenol-related proteins, MAFLD-associated targets, and hepatotoxicity-related genes (Figure 1A). This analysis revealed a subset of shared target proteins, indicating potential mechanistic intersections between Proliverenol bioactives and disease-associated pathways. Subsequently, protein–protein interaction (PPI) analysis using the STRING database demonstrated a highly interconnected interaction network (Figure 1B). The network displayed dense connectivity, suggesting coordinated biological regulation rather than isolated protein effects. This finding supports the hypothesis that Proliverenol acts through network-level modulation rather than single-target intervention.

### 2.3. Network Clustering and Functional Module Identification

**Figure 2:**
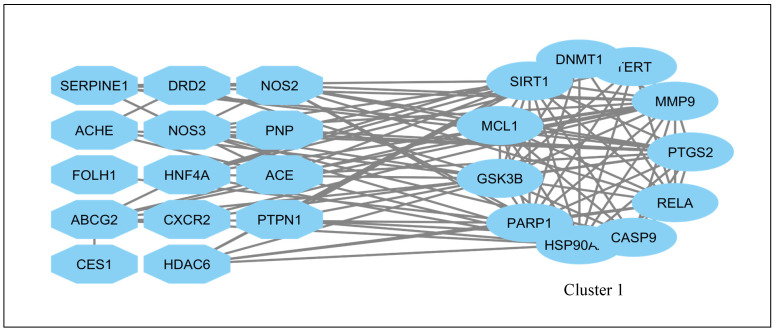
Protein–protein interaction network clustering of potential target proteins. Cluster 1 consists of 11 nodes and 49 edges.

The PPI network was further analyzed using MCODE clustering to identify highly interconnected functional modules. Cluster 1, identified as the most significant module, consisted of 11 nodes and 49 edges, indicating strong intramodular interactions (Figure 2). The high edge density within this cluster suggests a central regulatory role in disease-relevant signaling processes. Proteins within this cluster were predominantly associated with cell survival, inflammation, metabolic regulation, and stress-response pathways, which are central to MAFLD progression and hepatocellular injury.

### 2.4. Biological Process Enrichment and Functional Annotation

**Figure 3:**
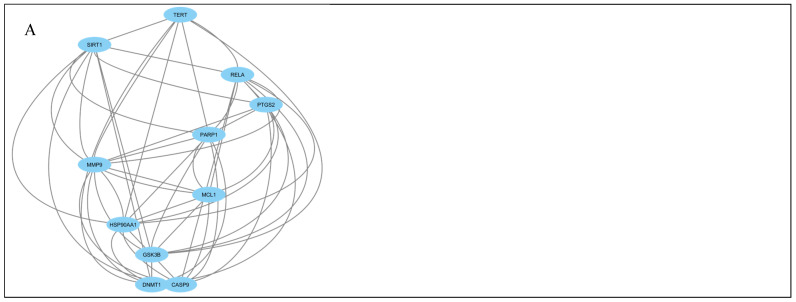

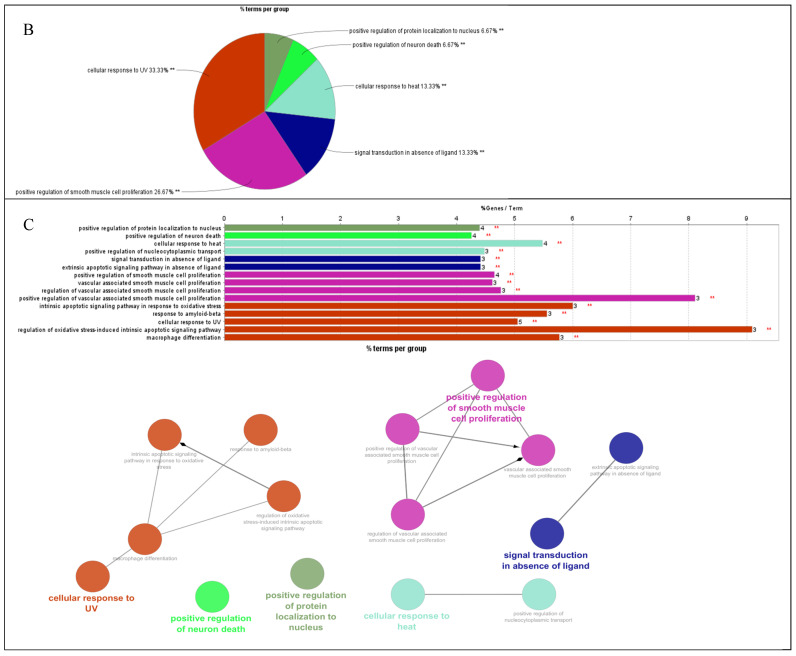
MCODE and ClueGO analysis, chosen cluster of module one (**A**), % term gene per group of biological processes (**B**), and biological processes of potential target protein clusters (**C**). ** indicates highly significant enrichment.

MCODE-derived Cluster 1 was subjected to ClueGO enrichment analysis to elucidate associated biological processes. The analysis demonstrated significant enrichment in processes related to regulation of apoptosis, inflammatory signaling, oxidative stress response, and lipid metabolic processes (Figure 3A–C). The percentage distribution of genes across biological process categories indicated a dominant contribution of pathways involved in cell death regulation and immune-mediated inflammation, reinforcing the relevance of this cluster to hepatoprotection and MAFLD pathophysiology.

### 2.5. KEGG Pathway Enrichment of Key Target Proteins

**Figure 4:**
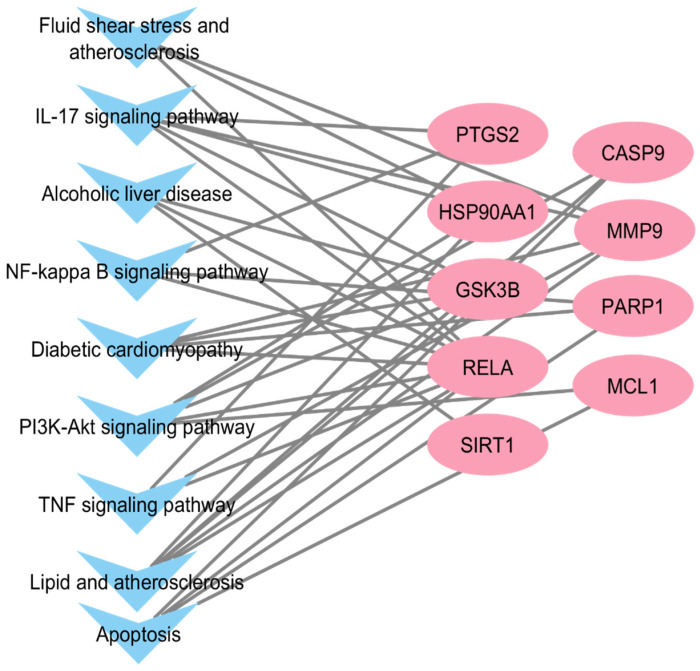
KEGG Pathways of Key Target Proteins.

KEGG pathway enrichment analysis revealed that the key target proteins were significantly associated with pathways involved in NF-κB signaling, insulin resistance, lipid metabolism, inflammatory mediator regulation, and cellular senescence (Figure 4). These pathways are well-recognized contributors to MAFLD development and progression, further supporting the mechanistic plausibility of Proliverenol as a multi-pathway modulator.

### 2.6. Gene Ontology Enrichment Analysis (Figure 5)

**Figure 5:**
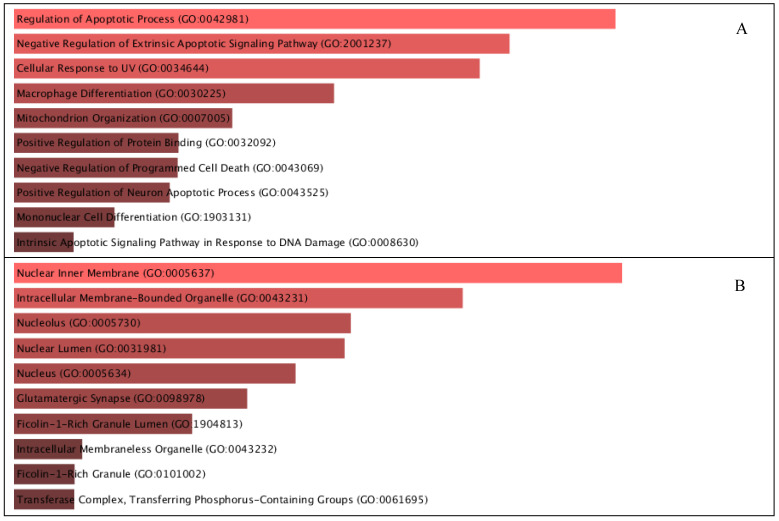

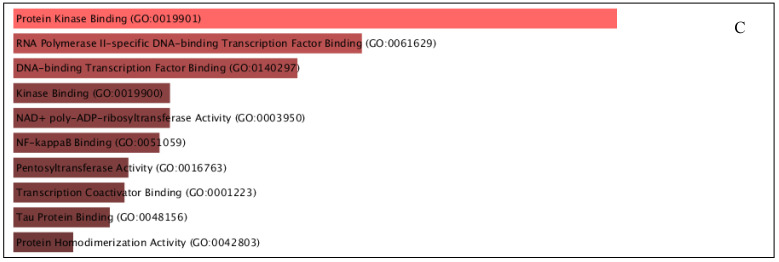
Gene Ontology of Biological Processes (**A**), Cellular Components (**B**), and Molecular Functions (**C**).

Gene Ontology (GO) analysis demonstrated significant enrichment across all three GO domains. In the Biological Process category, target proteins were enriched in processes related to response to oxidative stress, regulation of apoptosis, and inflammatory response (Figure 5A). The Cellular Component analysis indicated predominant localization within the cytosol, nucleus, and membrane-associated complexes, suggesting roles in transcriptional regulation and intracellular signaling (Figure 5B). Meanwhile, Molecular Function enrichment highlighted protein binding, enzyme regulatory activity, and transcription factor binding, reflecting the regulatory versatility of the identified targets (Figure 5C).

### 2.7. Molecular Docking Analysis of Proliverenol Compounds

Molecular docking analysis was performed to evaluate the binding affinity of Proliverenol-derived compounds against five key MAFLD-related targets from network pharmacology: MCL1, SIRT1, GSK3B, RELA, and PTGS2. Overall, multiple compounds exhibited strong binding affinities, comparable to or exceeding those of reference drugs (Table 2). Notably, cucumerin B, artoindonesianin P, and vitexin 2″-p-hydroxybenzoate demonstrated the most favorable binding energies across multiple targets, particularly against PTGS2, with binding affinities reaching −12.6 kcal/mol. Several compounds also showed strong interactions with SIRT1 and GSK3B, indicating potential roles in metabolic regulation and inflammation control. Compared with control ligands (metformin, pioglitazone, and silibinin from Silymarin), selected Proliverenol compounds exhibited comparable or superior binding affinities, supporting their potential relevance as multitarget modulators in MAFLD-associated pathways.

### 2.8. Comparative Docking Analysis with PTGS2

**Figure 6:**
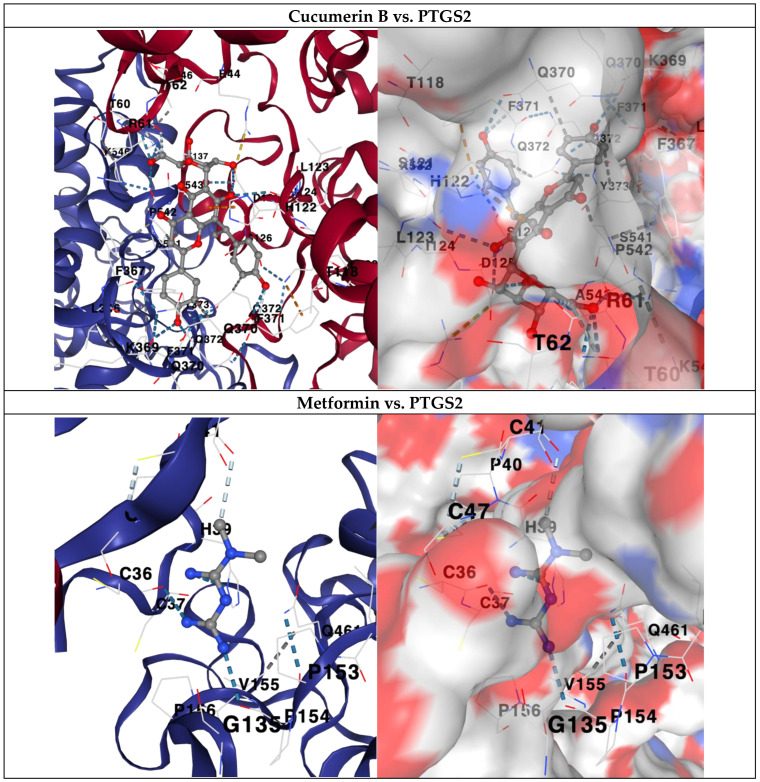

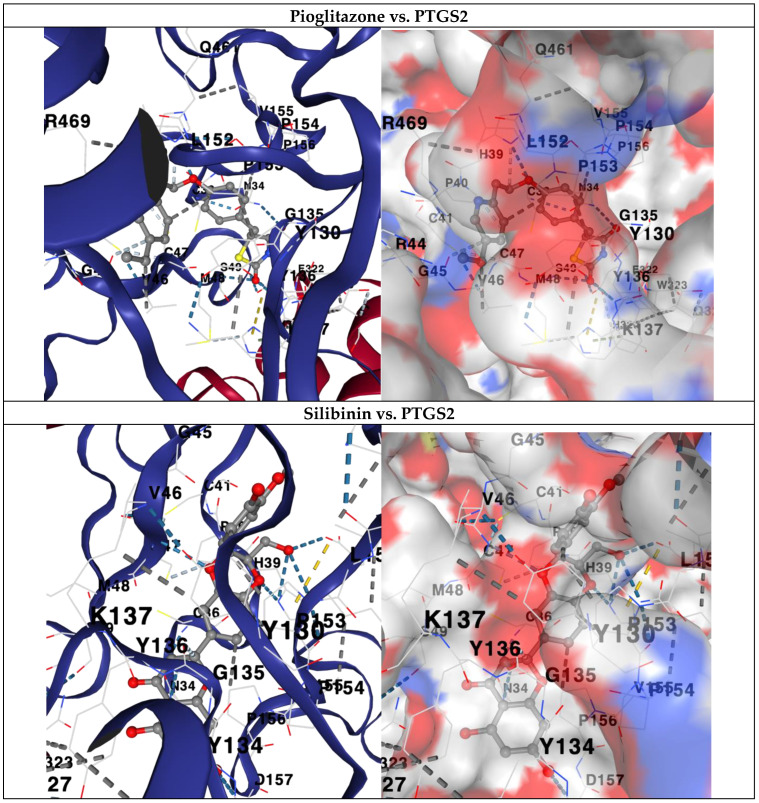
Molecular docking comparison of Proliverenol bioactive compounds and control drugs targeting PTGS2 (cyclooxygenase-2).

Further comparative docking analysis focusing on PTGS2 revealed that cucumerin B displayed stronger binding affinity than metformin and pioglitazone and was comparable to silibinin (Figure 6). This finding underscores the potential anti-inflammatory relevance of Proliverenol constituents through modulation of prostaglandin synthesis pathways.

## 3. Discussion

**Figure 7:**
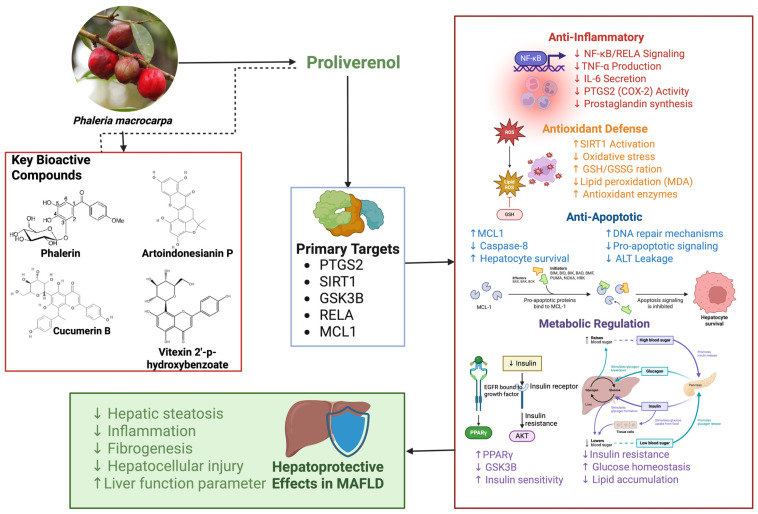
Multi-target hepatoprotective mechanisms of Proliverenol in MAFLD. Proposed mechanistic illustration of the hepatoprotective effects of Proliverenol (Veprolin™), a standardized fraction of *Phaleria macrocarpa* pericarpium, against metabolic dysfunction-associated fatty liver disease (MAFLD). Bioactive compounds identified via LC–HRMS analysis, including cucumerin B, artoindonesianin P, vitexin, vitexin 2″-p-hydroxybenzoate, and phalerin, were predicted to interact with key regulatory proteins (PTGS2, RELA, SIRT1, GSK3β, and MCL1) based on network pharmacology and molecular docking analyses. These protein–ligand interactions modulate multiple pathological pathways associated with MAFLD, including inflammatory signaling (NF-κB/COX-2), metabolic dysregulation, oxidative stress, and hepatocyte apoptosis. Collectively, these multi-target effects contribute to reduced hepatic inflammation, improved insulin sensitivity and lipid metabolism, attenuation of oxidative damage, enhanced hepatocyte survival, and overall hepatoprotection in MAFLD. Arrows indicate direction of effect. Upward arrows (↑) denote activation/upregulation, downward arrows (↓) denote inhibition/downregulation. This figure was created in BioRender. Nurkolis, F. (2026). https://BioRender.com/q0bbfmh (accessed on 19 February 2026).

The present study’s integrative analysis revealed that Proliverenol—a standardized bioactive extract of *P. macrocarpa*—contains 14 candidate phytochemicals that engage multiple MAFLD-related targets (Figure 7). Network pharmacology mapped these constituents to key NAFLD proteins (e.g., IL-6, PPARγ, NF-κB/RELA, TNFα) [21]. Molecular docking confirmed strong predicted binding affinities of several compounds to these targets [21]. Together, these results indicate a true “multi-target” profile for Proliverenol, in which its diverse constituents can potentially modulate the major signaling nodes of MAFLD (lipid metabolism, inflammation, oxidative stress, insulin signaling) simultaneously [21].

In the context of previously reported phytochemistry of *P. macrocarpa*, the composition identified in Proliverenol is largely consistent with known chemical characteristics of the fruit pericarp. Earlier studies have described the plant as being rich in phalerin, mangiferin, gallic acid, flavicordin-A, and various flavonoids and phenolic derivatives [15,22,23]. In our untargeted LC–HRMS/MS analysis, phalerin emerged as the most abundant constituent, confirming its role as a major marker compound of the species. However, certain phytochemicals previously reported in crude extracts (e.g., mangiferin or flavicordin-A) were not detected in the analyzed fraction. This difference is likely attributable to the fractionation and standardization processes used to obtain Proliverenol, which may selectively enrich specific polyphenolic and flavonoid components while reducing others. Therefore, the phytochemical profile observed in this study reflects the characteristics of a standardized bioactive fraction rather than the complete metabolite spectrum of the raw plant material. Importantly, the enrichment of flavonoids and phenolic derivatives in Proliverenol is mechanistically relevant, as these classes of compounds are widely recognized for their antioxidant, anti-inflammatory, and metabolic regulatory properties—biological activities that align closely with the multi-target network effects predicted in our pharmacoinformatics analysis.

Mechanistically, the data suggest that Proliverenol exerts hepatoprotection by dampening inflammation and oxidative injury and by improving metabolic signaling. In particular, many of the predicted targets lie in the NF-κB/TNFα/IL-6 axis—a central driver of hepatic inflammation—as well as in pathways regulating insulin sensitivity and antioxidant defenses. For example, NF-κB (RELA) is activated by AKT1/TNFα and in turn induces pro-inflammatory cytokines (TNFα, IL-6) [24,25]. Docking and network results indicate Proliverenol’s flavonoids can bind to RELA and PTGS2, implying inhibition of NF-κB signaling. Consistent with this, previous in vivo work showed *P. macrocarpa* extract (Proliverenol) lowered TNF-α and TGF-β1 levels and reduced lipid peroxidation (MDA) while raising GSH/GSSG in a CCl_4_ liver injury model [18]. These biochemical changes reflect reduced inflammation and oxidative stress. Similarly, predicted engagement of PPARγ (a regulator of lipid metabolism and insulin sensitivity) suggests Proliverenol may alleviate insulin resistance; PPARγ activation is known to improve insulin signaling and also possesses anti-inflammatory/anti-fibrotic effects in the liver [21]. In sum, Proliverenol’s constituents appear to interrupt the feed-forward cycle of fatty liver: they inhibit NF-κB-driven cytokine release, scavenge ROS, and modulate lipid/glucose regulators, thereby protecting hepatocytes and stellate cells from MAFLD pathology [18,24].

These findings are in broad agreement with prior literature on natural multi-target therapies for fatty liver. Many plant polyphenols (quercetin, kaempferol, luteolin, etc.) have documented anti-steatotic, anti-inflammatory, and insulin-sensitizing effects in NAFLD models [21,26]. For instance, network pharmacology studies of herbal formulations (e.g., Danning Tablet) likewise identified IL-6, TNFα, PPARs and NF-κB as hubs and reported modulation of lipid metabolism, inflammation, oxidative stress and insulin resistance [21]. This “multi-hit” concept—that complex diseases require multi-component interventions—has growing support. A recent review noted that natural products can simultaneously target lipid handling, IR and inflammatory/oxidative pathways in NAFLD [26]. Our results extend these observations to *Phaleria* bioactives. Notably, the specific targets (RELA, PPARG, CASP3, etc.) and pathways (inflammation, apoptosis, fibrosis) predicted here mirror those implicated in other phytochemical studies [21]. Thus, Proliverenol’s action is congruent with the known pleiotropic actions of many hepatoprotective natural compounds.

Clinically, the multi-target profile of Proliverenol suggests it may be a promising candidate for MAFLD therapy. There is currently no approved pharmacotherapy for NAFLD/MAFLD, and patients often receive only lifestyle advice and off-label supplements [21]. In this context, botanical extracts that broadly modulate metabolism and inflammation are of great interest. Indeed, agents like silymarin and berberine are under active clinical investigation for NAFLD [21,26]. Proliverenol—a standardized fraction already shown to protect the liver in other models—could similarly confer benefit via its combined actions on fat accumulation, insulin signaling and inflammatory/fibrotic pathways. If validated, a plant-derived multi-component therapy might improve biochemical markers (ALT/AST, lipids), reduce liver fat/inflammation, and prevent fibrosis. However, translating these findings will require careful formulation: standardization of Proliverenol, determination of effective dosing, and assessment of safety and drug interactions in MAFLD patients.

This study has important limitations. All conclusions are based on in silico methods (untargeted metabolite profiling, network analysis, docking) and literature inference. Although we identified 14 putative bioactives, their structures and concentrations must be confirmed by targeted analysis (e.g., LC–MS/MS with standards or NMR). Network predictions rely on database targets and did not account for pharmacokinetics or synergistic effects. Similarly, docking indicates only binding potential; it does not prove functional inhibition or selectivity. A direct comparison with proprietary commercial reference standards could not be performed due to restricted access to full compositional disclosure and reference spectral libraries. Nevertheless, compound annotation reliability was ensured through high-resolution MS/MS spectral matching, accurate mass constraints, isotopic pattern evaluation, and stringent similarity score thresholds, consistent with MSI Level 2 identification criteria. Thus, our mechanistic hypotheses, while plausible and consistent with animal data [18], require experimental validation. Future work should include in vitro assays (e.g., cytokine release, lipid accumulation in hepatocytes) and in vivo MAFLD models to test Proliverenol’s efficacy and mechanisms. In short, although molecular docking bolsters our proposed interactions, experimental validations are still needed to confirm Proliverenol’s hepatoprotective, anti-inflammatory, and metabolic effects [21].

Our findings are supported by prior network pharmacology research in NAFLD [21], phytochemical hepatoprotection studies [18], and reviews of natural products in NAFLD [21,24]. Detailed docking results and target analyses were drawn from network modeling of known NAFLD targets [21]. All cited studies provide context for Proliverenol’s multi-target mechanism and highlight the need for further validation.

## 4. Materials and Methods

### 4.1. Sample Preparation

Proliverenol (Veprolin™) is a standardized bioactive fraction derived from *P. macrocarpa.* Each tablet contains 500 mg of Proliverenol, corresponding to a dry extract of *P. macrocarpa* pericarpium (fruit pericarp). The herbal preparation is obtained from *P. macrocarpa*. The plant material originated from Central Java, Indonesia, and was taxonomically identified by Herbarium Bogoriense, Research Center for Biology, Indonesian Institute of Sciences, with certificate No. 1261/IPH.1.02/If.8/XII/2009.

The extract was prepared by maceration of sliced dried fruit pericarp using a water-based extraction solvent. The resulting extract was filtered, concentrated, oven-dried, and stored in a tightly closed container. The drug-to-extract ratio (DER) and genuine extract proportion range are consistent with the manufacturer’s standardized quality specifications. Veprolin™ was obtained from PT Dexa Medica (Cikarang, Indonesia), where it is commercially produced under established quality control and standardized phytochemical profiling.

For metabolomic analysis, 10 mg of Veprolin™ powder was accurately weighed and extracted with 1 mL of 90% methanol (HPLC grade; Fisher Chemicals, Waltham, MA, USA). The mixture was homogenized using vortex agitation at 3000 rpm for 1 min to ensure complete dissolution. The extract was subsequently filtered through a 0.20 µm nylon membrane filter and transferred into HPLC vials prior to LC–HRMS/MS analysis. Each sample was prepared and analyzed in duplicate (two independent analytical runs) to ensure analytical reproducibility and data reliability.

Methanol (HPLC grade) was purchased from Fisher Chemicals (Waltham, MA, USA). Ultrapure water was obtained from a Milli-Q purification system (MilliporeSigma, Burlington, MA, USA). Nylon membrane syringe filters (0.20 µm) were sourced from Merck (Merck KGaA, Darmstadt, Germany).

### 4.2. Untargeted Metabolomic Analysis

Untargeted metabolomic analysis was performed using liquid chromatography–high-resolution mass spectrometry (LC–HRMS) [27,28]. Untargeted metabolomics was intentionally employed to complement existing standardized phytochemical profiling by providing a holistic characterization of the formulation’s metabolite composition.

Chromatographic separation was carried out using a Thermo Scientific™ Vanquish™ Horizon UHPLC system equipped (Thermo Scientific, Waltham, MA, USA) with a binary pump and an Accucore™ C18 analytical column (100 mm × 2.1 mm, 2.6 µm). The mobile phase consisted of (A) MS-grade water containing 0.1% formic acid and (B) MS-grade acetonitrile containing 0.1% formic acid, delivered at a flow rate of 0.3 mL/min. A linear gradient was applied starting from 5% B, increasing to 90% B over 16 min, maintained at 90% B for 4 min, and returned to 5% B until a total runtime of 25 min. The column temperature was maintained at 40 °C, and the injection volume was set at 5 µL.

Mass spectrometric detection was performed using a Thermo Scientific™ Orbitrap™ Exploris™ 240 high-resolution mass spectrometer operated in full MS/dd-MS^2^ mode with polarity switching (positive and negative ionization). Full MS spectra were acquired at a resolution of 60,000 FWHM over a scan range of *m*/*z* 70–800, with a maximum injection time of 100 ms and a mass tolerance of 5 ppm. Data-dependent MS^2^ acquisition was conducted at a resolution of 30,000 FWHM using stepped normalized collision energies of 30, 50, and 70, with nitrogen as the collision gas.

Ionization was achieved using an OptaMax™ NG heated electrospray ionization (H-ESI) source (Thermo Scientific, Waltham, MA, USA), operated at spray voltages of 3500 V (positive) and 2500 V (negative). The sheath, auxiliary, and sweep gas flows were set at 35, 7, and 1 arbitrary units, respectively. The ion transfer tube temperature was maintained at 300 °C, while the vaporizer temperature was set to 320 °C.

Raw LC–HRMS data from duplicate analyses were processed using Thermo Scientific™ Compound Discoverer™ version 3.3. Metabolite annotation was performed by matching accurate mass, isotopic pattern, and MS^2^ fragmentation spectra against multiple spectral libraries and databases, including mzCloud (endogenous metabolites, natural products, medicines, toxins, steroids, vitamins, and hormones), ChemSpider-supported databases (NPAtlas, Phenol-Explorer, PubChem, Springer Nature), and curated mass list databases such as LipidMaps, Arita Lab flavonoid database, and Endogenous Metabolites database.

### 4.3. In Silico Studies

#### 4.3.1. Target Fishing

The identification of potential target proteins for each compound was conducted using the TargetNet database (https://targetnet.scbdd.com/, accessed on 19 February 2026) with a probability threshold greater than 0.6 [29]. Subsequently, disease-related target proteins were retrieved from the OMIM (https://www.omim.org/, accessed on 16 December 2025), NCBI (https://www.ncbi.nlm.nih.gov/, accessed on 19 February 2026), and GeneCards databases (https://www.genecards.org/, accessed on 16 December 2025) [30]. All identified targets were then integrated, and duplicate entries were removed. Finally, a Venn diagram analysis was performed using Venny 2.1.0 to determine the overlapping targets (https://bioinfogp.cnb.csic.es/tools/venny/, accessed on 16 December 2025).

#### 4.3.2. Network Analysis

Following the Venn diagram analysis, the potential target proteins were further analyzed for protein–protein interactions (PPIs) using the STRING database (https://string-db.org/, accessed on 16 December 2025) [31]. The resulting PPI network was then subjected to cluster analysis using the MCODE algorithm to identify significant protein modules. These modules were subsequently analyzed using ClueGO to elucidate biological process-related pathways closely associated with hepatotoxicity and metabolic dysfunction-associated fatty liver disease (MAFLD). KEGG pathway enrichment analysis was performed using the DAVID database (https://davidbioinformatics.nih.gov/, accessed on 16 December 2025), with pathways selected based on a *p*-value < 0.05. Gene Ontology (GO) enrichment analysis, including Biological Processes, Molecular Functions, and Cellular Components, was conducted using the Enrichr platform. Finally, five potential target proteins with strong regulatory roles or close associations with MAFLD and hepatotoxicity were selected for molecular docking analysis [32].

#### 4.3.3. Molecular Docking Analysis

The three-dimensional structures of each compound in SDF format were obtained from the PubChem database (https://pubchem.ncbi.nlm.nih.gov/, accessed on 18 December 2025). Meanwhile, the five key target proteins (MCL1, SIRT1, GSK3B, RELA, and PTGS2) were downloaded in PDB format from the RCSB Protein Data Bank (https://www.rcsb.org/, accessed on 18 December 2025). Molecular docking analysis between each test compound and the target proteins was subsequently performed using the CB-Dock2 platform. The lowest binding affinity value was selected, as it indicates a stronger interaction between the test compound and the target protein [33,34,35].

## 5. Conclusions

This integrative study provides comprehensive mechanistic insights into the hepatoprotective potential of Proliverenol in metabolic dysfunction-associated fatty liver disease. Untargeted metabolomic profiling confirmed that Proliverenol comprises a diverse array of bioactive phytochemicals, predominantly flavonoids and phenolic compounds, which collectively contribute to its multitarget biological activity. Network pharmacology analysis revealed that Proliverenol-associated targets intersect extensively with MAFLD- and hepatotoxicity-related proteins, forming a densely connected protein–protein interaction network that underscores a systems-level mode of action rather than a single-target effect.

Functional enrichment analyses highlighted key biological processes and signaling pathways involved in apoptosis regulation, inflammatory responses, oxidative stress control, lipid metabolism, insulin resistance, and cellular senescence—hallmark mechanisms underlying MAFLD pathogenesis. Molecular docking further validated these findings by demonstrating strong and stable interactions between major Proliverenol constituents and critical regulatory proteins such as PTGS2, SIRT1, GSK3B, RELA, and MCL1, with binding affinities comparable to established therapeutic agents.

Collectively, these results support the concept that Proliverenol acts as a multi-target modulator capable of simultaneously attenuating inflammation, improving metabolic signaling, and protecting hepatocytes from injury. This systems pharmacology-based evidence strengthens the scientific rationale for the development of Proliverenol as a mechanism-driven hepatoprotective intervention for MAFLD. Nevertheless, the conclusions are based predominantly on computational predictions and indirect mechanistic inference. The absence of direct experimental validation constitutes a key limitation of this study. Therefore, further experimental investigations, including targeted phytochemical quantification, in vitro functional assays, and in vivo MAFLD models, are necessary to confirm the predicted biological activities. Subsequent clinical studies will be essential to establish therapeutic efficacy, safety, and translational applicability.

## Figures and Tables

**Table 1:** Phytochemical constituents identified in Proliverenol via HPLC-ESI-HRMS/MS analysis.

Compounds	Formula	Area (×10^7^, Mean ± SD)	Annot. DeltaMass [ppm]	RT (Minutes)	*mz*	Reference Ion
Phalerin	C_20_H_22_O_10_	458.50 ± 6.68	−0.685 ± 0.1202	3.2315 ± 0.0064	421.1137 ± 1 × 10^−4^	[M−H]^−1^
Phenol	C_6_H_6_O	4.94 ± 0.08	−0.205 ± 0.2333	2.187 ± 0.0014	93.0346 ± 0	[M−H]^−1^
Adenosine	C_10_H_13_N_5_O_4_	62.40 ± 0.81	−0.61 ± 0.2404	0.872 ± 0	268.1039 ± 1 × 10^−4^	[M+H]^+1^
Catechol	C_6_H_6_O_2_	3.23 ± 0.04	0.175 ± 0.1485	1.5805 ± 0.0064	109.0295 ± 0	[M−H]^−1^
4-Oxoproline	C_5_H_7_NO_3_	8.95 ± 0.83	−0.28 ± 0.0424	0.873 ± 0.0014	130.0497 ± 0	[M+H]^+1^
Kynurenic acid	C_10_H_7_NO_3_	4.50 ± 0.10	−0.63 ± 0.1697	2.1835 ± 0.0035	190.0498 ± 0	[M+H]^+1^
Gluconic acid	C_6_H_12_O_7_	7.09 ± 0.23	0.23 ± 0	0.67 ± 0.0014	195.0511 ± 0	[M−H]^−1^
Azelaic acid	C_9_H_16_O_4_	12.42 ± 0.28	0.635 ± 0.1202	4.829 ± 0	187.0977 ± 0	[M−H]^−1^
Succinic acid	C_4_H_6_O_4_	9.36 ± 0.01	0.15 ± 0.0141	0.904 ± 0	117.0194 ± 0	[M−H]^−1^
Vitexin	C_21_H_20_O_10_	1.19 ± 0.07	−0.06 ± 0.2828	3.8785 ± 0.0035	433.1128 ± 2 × 10^−4^	[M+H]^+1^
(-)-Caryophyllene oxide	C_15_H_24_O	1.96 ± 0.62	−0.545 ± 0.1061	6.5705 ± 0.1732	221.1899 ± 0	[M+H]^+1^
Vitexin 2″-p-hydroxybenzoate	C_28_H_24_O_12_	10.82 ± 0.28	−0.07 ± 0.099	7.003 ± 0.0028	551.1197 ± 1 × 10^−4^	[M−H]^−1^
Artoindonesianin P	C_20_H_16_O_7_	0.77 ± 0.76	−0.775 ± 0.3182	6.764 ± 1.1045	368.0893 ± 1.4244	[M+H]^+1^
Cucumerin B	C_29_H_28_O_11_	0.84 ± 0.59	−0.19 ± 0.6788	7.1905 ± 0.333	551.1559 ± 1 × 10^−4^	[M−H]^−1^

**Table 2:** Molecular docking binding affinities (kcal/mol) of Proliverenol compounds and control ligand against key MAFLD targets.

Compounds	MCL1 (5KU9)	SIRT1 (4I5I)	GSK3B (1I09)	RELA (1NFI)	PTGS2 (5KIR)
Phalerin	−7.5	−9.5	−8.3	−8.6	−9.5
Phenol	−4.7	−5.3	−4.7	−5.1	−5.0
Adenosine	−6.6	−8.1	−6.6	−8.0	−9.0
Catechol	−5.2	−5.6	−5.2	−5.3	−5.5
4-Oxoproline	−5.5	−5.6	−5.1	−5.6	−5.4
Kynurenic acid	−6.9	−7.8	−6.8	−8.0	−7.9
Gluconic acid	−5.5	−6.1	−6.5	−5.8	−6.1
Azelaic acid	−5.7	−6.2	−6.3	−5.6	−6.1
Succinic acid	−4.6	−5.1	−4.8	−5.0	−4.9
Vitexin	−6.8	−8.2	−8.4	−8.4	−9.5
(-)-Caryophyllene oxide	−5.2	−7.2	−6.7	−6.0	−7.0
Vitexin 2”-p-hydroxybenzoate	−7.6	−8.6	−9.2	−8.7	−10.9
Artoindonesianin P	−8.3	−10.1	−9.1	−9.1	−11.1
Cucumerin B	−9.4	−8.6	−8.7	−8.6	−12.6
Metformin	−5.2	−5.2	−5.7	−5.7	−7.9
Pioglitazone	−9.2	−10.4	−7.7	−8.8	−9.0
Silibinin	−7.7	−10.1	−9.2	−9.8	−11.9

## Data Availability

The data presented in this study are available on request from the corresponding author due to intellectual property considerations and ongoing analyses.
